# Prevalence and causes of blindness, visual impairment among different ethnical minority groups in Xinjiang Uygur autonomous region, China

**DOI:** 10.1186/s12886-018-0705-6

**Published:** 2018-02-13

**Authors:** Yanping Li, Wenyong Huang, Aoyun Qiqige, Hongwei Zhang, Ling Jin, Pula Ti, Jennifer Yip, Baixiang Xiao

**Affiliations:** 1 The Affiliated Eye Hospital of Nanchang University, Nanchang City, China; 2 State Key Laboratory of Ophthalmology, Zhongshan Ophthalmic Centre, Sun Yat-sen University, #54 South Xianlie Road, Guangzhou City, Guangdong Province 510060 China; 3 Tacheng District People’s Hospital, Tacheng City, Xinjiang Uygur Autonomous Region China; 4 Altay District People’s Hospital, Altay City, Xinjiang Uygur Autonomous Region China; 5 Xinjiang Regional Hospital, Urumqi, China; 6 International Center for Eye Health, London School of Hygiene & Tropical Medicine, London, UK

**Keywords:** Ethnical minority groups, Prevalence of blindness, Cataract surgical coverage, Eye services, Equity, Xinjiang, China

## Abstract

**Background:**

The aim of this cross-sectional study is to ascertain the prevalence and causes of blindness, visual impairment, uptake of cataract surgery among different ethnic groups in Xinjiang Uygur Autonomous Region, China.

**Methods:**

Four thousand one hundred fifty people at 50 years and above from different minority ethnic groups were randomly selected for an eye examination. The four trained eye teams collected data using tumbling E visual chart, torch, portable slit lamp and direct ophthalmoscope in 2015. The World Health Organization’s definition of blindness and visual impairment (VI) was used to classify patients in each ethnic group. Data were analyzed by different minority groups and were compared with Han Chinese.

**Results:**

3977 (95.8%) out of 4150 people were examined. The prevalence of blindness from the study population was 1.7% (95% confidence interval: 1.3–2.2%).There was no significant difference in prevalence of blindness between Han Chinese and people of Khazak and other minority ethnic groups, nor, between male and female. Cataract was the leading course (65.5%) of blindness and uncorrected refractive error was the most common cause of VI (36.3%) followed by myopic retinopathy. The most common barrier to cataract surgery was lack of awareness of service availability.

**Conclusions:**

This study documented a low blindness prevalence among people aged 50 years and over comparing to prevalence identified through studies of other regions in China. It still indicates blindness and un-operated cataract as the significant public health issue, with no evidence of eye health inequalities, but some inequities in accessing to cataract surgery amongst ethnic minority groups in Xinjiang.

## Background

The joint global initiative of the International Association of Prevention of Blindness (IAPB) and the World Health Organization (WHO) has set targets to help global efforts to eliminate avoidable blindness [1]. The most recent initiative aims to reduce eye health inequalities through equitable access to blindness-prevention services for people of different gender, and ethnicity [2].

In line with the Vision 2020 initiative, there has been an increase in the number of effective programs and policies to prevent blindness in China. Consequently, the national annual cataract surgical rate (CSR) has increased from under 800 patients per million population in 2009 to 1400 in 2015 [3]. Studies have been conducted to document the prevalence of blindness in adults aged 50 years and older, with recent figures ranging from 1.27% to 5.46% [4]. However, to date there have been no studies on the prevalence of blindness or visual impairment, and rate of ocular service uptake in Xinjiang Uygur Autonomous Region (Xinjiang), which is home to the largest proportion of minority ethnic groups in China. Economic and health inequalities have been documented in China, but little is known about eye health inequalities. Findings from this study can help address this gap in knowledge and lead to programs designed to ensure equitable access to eye care for people of ethnic minorities in China.

**Fig. 1:**
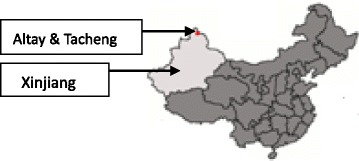
Map of China. The map picture was created by the user “Quigley” under the Creative Code “CC BY-SA 3.0” at the wiki website of https://en.wikipedia.org/wiki/Xinjiang. It has been credited and edited with study sites being marked

Xinjiang is located in the northwest of China (Fig. 1) and shares borders internationally with Mongolia, Russia, Khazakhstan, Kyrgyzstan, Tajikistan, Pakistan, India, Afghanistan, and domestically with Gansu and Qinghai Provinces and the Tibet. The region consists of 14 prefectures (the administration system between province and county) which are further divided into 88 counties. As of 2013 this area was home to 22.33 million people, with the majority consisting of Uygur (43.6%) and Han Chinese (40.6%), followed by Khazaks (8.3%) and other ethnic groups (7.5%) including Hui, Kirgiz, Mongolian, Tajik and Ozbek [5]. Although assimilation among different ethnic groups is very common in the modern life, Uyghur and Khazaks people are still more nomadic, living more spread and facing less tensive working hours, comments from the local people in Xinjiang.

Access to even basic eye services can be difficult for this population. People live in dispersed communities and generally travel by motorbike or horse to small towns where they can take a bus to reach prefecture hospitals, which can be over 6 h away. At prefecture where examination on eyes including retina, cataract operation and refractive errors services are routinely supplied by the local eye doctors or rarely by visiting ophthalmologists from provincial level.

There is little information on the uptake of ocular health services by various ethnic groups in Xinjiang. This study examined the prevalence and causes of visual impairment, blindness, and cataract surgery uptake by various ethnic minorities (Uygur, Khazak, and Other) compared to the local Han Chinese, as well as the possible effect that distance to ocular services has on eye health.

## Method

Based on census data in 2014, 123,704 (23.3%) people were aged 50 years and older in Altay, and 852,379 people (22.6%) aged 50 years old and above in Tacheng. The two areas are adjacent and share similar population composition and health economic situations. They were treated as a single study site. This part of Xinjiang had lower proportions of Uyghur people compared to South of the Region [6] and higher proportions of Khazak. The International Non-government Organization planned to have a three-year eye care program in these two areas and the study was designed for baseline data collection to guide the program there.

The Rapid Assessment of Avoidable Blindness (RAAB6.0) [7] software was used for sample size calculation and determining our sampling frame. Based on previous studies in China and discussion with local doctors, we estimated a prevalence of blindness of 2.4% in people aged 50 and above. Based on 25% precision, and estimated non-response rate of 15%, 95% confidence intervals, and design effect of 1.5, we determined that the sample size of 4150 were required, which would be recruited in clusters of 50 people aged 50 years or older in Altay and Tacheng. Clusters were selected using probability proportional to size sampling with list of villages as the sampling frame.

Four survey teams, each consisting of an ophthalmologist, a nurse and a refractionist, were trained for 1 week by a qualified study trainer (BX) in June 2015. There was good Inter-observation agreement between teams, with kappa co-efficient greater than 0.6.There was a doctor or nurse of an ethnic minority (Khazak in Altay and Uygur in Tacheng) who could communicate with subjects in the minority dialect in each team.

We selected households in each village using compact segment sampling [8]. The team went house to house to collect data from eligible adults in the selected segments of approximately 83 older people. To optimize the number of subjects encountered on the data collection, village heads were notified from a meeting 1 week and again 1 day via phone calls prior to the scheduled visit and asked to inform residents of the upcoming site visit.

### Ophthalmic examination

Study subjects were initially requested on whether having distance or reading glasses, surgical and traumatic history after oral agreement to the study obtained. Presenting visual acuity (PVA) was measured using two tumbling E-charts. One with size of 6/12 and the other with size 6/18 on one side and 6/60 on the other side at 6 m or 3 m, so that attainment of VA of 6/12, 6/18, 6/60 and 3/60 could be assessed.VA was tested outside of the house in full day light. If VA was below 6/18 in either eye, pinhole was then used as a proxy for best corrected VA (BCVA) and if VA could be improved to 6/18, refractive errors were defined as the cause of VA. All the subjects were examined by the ophthalmologist in the house using a torch, direct ophthalmoscope and/or portable slit lamp to detect the causes of visual impairment. The pupil was dilated for diagnosis if the examiner was unable to determine primary cause of visual impairment without dilation. Study subject with VA less than 6/18 in either eye had the principal cause of the visual impairment/blindness diagnosed. Blindness was defined as VA worse than 3/60 in the better eye with available spectacle correction, severe visual impairment (SVI) as VA worse than 6/60–3/60, moderate visual impairment (MVI) as VA below 6/18–6/60, and early visual impairment (EVI) as VA below 6/18–6/12. People with MVI or worse from cataract were asked why they had not sought for surgery, and people with aphakia or pseudophakia were questioned about the details of their cataract surgery.

If a subject was not present for the team visit, a second visit was booked with other family members, or neighbors, or by a call to the subject’s mobile phone. If the subject was not present at the second home visit, s/he was treated as “missed” and eye health history was taken from a family member, neighbors or via mobile phone call to the subject. The RAAB study form [8] were used for data collection, including basic information on each study subject, medical history on eyes, whether wearing glasses and which glasses were included. Team members input data on the same day as they completed collecting information from a cluster (50 subjects). Data collection was completed within 3 months of the start date in 2015.

The Chi-squared or Fisher-exact test was used for comparisons of study indicators between a) minority groups and Han Chinese, b) males and females, and c) pair-wise comparisons between distance-to-eye-service categories. The cataract surgical coverage by person was calculated using the formula developed by Limburg, H (1989) [9], which is the number of persons operated on divided by the total number of people with cataract, including operated and non-operated persons in a designated geographical area. Statistic analyses were performed using STATA13.1 (Stata Corp, College Station TX, USA).

Ethical approval was obtained from the medical ethical committee in Xinjiang Regional Hospital. Informed consent was obtained from all eligible participants. All the eye patients found in the study were referred to Altay and Tacheng Hospitals for treatment or free cataract operations, supported by the local hospitals and medical insurance.

## Results

Data was collected from 3977 of 4150 eligible participants (95.8%). Table 1 shows the participation rates amongst different minority ethnic groups in the study. Khazaks were more likely to be unavailable for examination due to nomadic lifestyle. Among the 3977 people examined, 1632 (41.0%) were 50–59 years old; 749 (34.5%), 387 (17.9%), 127 (5.9%) were in the 60–69, 70–79, and over 80 years old age groups, respectively. Distributions of ethnic minorities and gender in the study population were comparable to the source population (Table 1).

**Table 1:** Subjects by ethnic group, and examination status, by age and gender

Ethnicity	Population in study areas		People selected		People examined		Not available		Refused		Unable		Total examined	
	n	%	n	%	n	%	n	%	n	%	n	%	n	%
Han	641,783	(46.2)	1775	42.8	1712	(96.5)	45	(2.5)	8	(0.5)	10	(0.6)	1712	100
Kazak	551,318	(39.7)	1837	(44.3)*	1747	(95.1)	80	(4.4)**	6	(0.3)	4	(0.2)	1747	100
Yugur	51,467	(3.7)	108	(2.6)	105	(97.2)	3	(2.8)	0	0.0	0	0.0	105	100
Other	145,862	(10.5)	430	(10.4)	413	(96.1)	11	(2.6)	2	(0.5)	4	(I0.9)	413	100
Total	1,390,430	(100)	4150	(100)	3977	(95.8)	139	(3.4)	16	(0.4)	18	(0.4)	3977	100
	Male				Female				Total					
Age	Population in study areas		People Examined		Population in study areas		People Examined		Population in study areas		People Examined			
	n	%	n	%	n	%	n	%	n	%	n	%		
50–59 years	11,771	45.4	727	40.2	11,730	44.1	905	41.7	23,501	44.8	1632	41.0		
60–69 years	8495	32.8	585	32.3	8822	33.2	749	34.5	17,317	33.0	1334	33.5		
70–79 years	4675	18.0	381	21.1	5020	18.9	387	17.9	9695	18.5	768	19.3		
80+ years	985	3.8	116	6.4	1012	3.8	127	5.9	1997	3.8	243	6.1		
Total	25,926	100	1809	100	26,584	100	2168	100	52,510	100	3977	100		

*It seemed we selected less Han Chinese and more Khazak compared their proportion of total population in study areas (*p* < 0.5)

**p < 0.01 Khazak people had highest rate of “not available” as they were mostly nomadic

Overall, there were 69 blind people (1.7%, 95% Confidence Interval -CI 1.3%–2.2%) with PVA below 3/60 in both eyes in this study population (Table 2). The gender and age adjusted blindness prevalence was 1.3% (95% CI: 0.8–1.7%) (Table 2). People of Khazakh origin had a higher prevalence of blindness (2.3%, 95% CI:1.7–3.1), though this was not statistically significant. The prevalence of SVI, MVI, EVI among examined people was 1.2%, 3.6%, 5.7% respectively. There was no significant difference in the prevalence of severe or moderate visual impairment between men and women, or between different minority ethnic groups (Table 2). However, higher proportion of Han Chinese had EVI (7.4%, 95%CI = 6.2–8.7%) compared to people of Khazak origin (4.1%, 95%CI = 3.3–5.2%).

**Table 2:** Sample and adjusted prevalence of blindness, severe (SVI), moderate (MVI) and early (EVI) visual impairment - bilateral PVA

Sample results											Age and Gender Adjusted results					
	Han		Kazak		Uygur		Other		Total		Males		Females		Total	
	n	% (95% CI)	n	% (95% CI)	n	% (95% CI)	n	% (95% CI)	n	% (95% CI)	n	% (95% CI)	n	% (95% CI)	n	% (95% CI)
Blindness	27	1.6 (1.1–2.3)	40	2.3 (1.7–3.1)	0	0	2	0.5 (0.1–1.9)	69	1.7 (1.4–2.2)	1976	1.3 (0.7–1.8)	2033	1.3 (0.8–1.7)	4009	1.3 (0.8–1.7)
Severe VI	20	1.2 (0.8–1.8)	16	0.9 (0.6–1.5)	3	2.9 (0.9–8.5)	10	2.4 (1.3–4.4)	49	1.2 (0.9–1.6)	1787	1.1 (0. 5–1.7)	1551	1.0 (0.5–1.4)	3338	1.1 (0.7–1.4)
Moderate VI	70	4.1 (3.2–5.1)	50	2.9 (2.2–3.8)	7	6.7 (3.2–13.4)	16	3.9 (2.4–6.2)	143	3.6 (3.1–4.2)	4701	3.0 (2.2–3.8)	5000	3.1 (2.3–3.9)	9701	3.1 (2.4–3.7)
Early VI	126	7.4 (6.2–8.7)	72	4.1 (3.3–5.2)*	4	3.8 (1.4–9.8)	24	5.8 (3.9–8.5)	226	5.7 (5.0–6.4)	6478	4.2 (3.0–5.3)	9392	5.8 (4.7–7.0)	15,870	5.0 (4.0–6.0)

*EVI prevalence of Khazak people is significantly lower than that of Han Chinese * *P* < 0.01

The sample prevalence of blindness, severe, moderate and early visual impairment were used to extrapolate the number of people at 50 years and above with bilateral blindness, severe visual impairment, moderate and early visual impairment. Results indicate that an estimated 4562 blind people live in Altay and Tacheng consisting of Khazak (32.1%), Han (31.5%), Uygur (1.9%) and other ethnic minorities (7.5%). We also estimated that there are 3336, 9696 and 15,868 people with SVI, MVI and EVI, respectively.

The main cause of blindness was untreated cataract followed by myopic retinopathy and other posterior segment diseases (Table 3). Uncorrected refractive error (URE) was the main cause of EVI and MVI, followed by cataract, and age-related macular degeneration. In total, among all causes of VI, 66.7% were treatable and 87% were avoidable.

**Table 3:** Principal cause of blindness, Severe Visual Impairment (VI), Moderate (MVI) and Early Visual Impairment (EVI) in persons

	Blindness	Severe VI	Moderate VI	Early VI
	%	%	%	%
1. Uncorrected Refractive Error	1.4%	16.3%	23.8%	49.1%
3. Cataract untreated	65.2%	34.7%	34.3%	23.0%
4. Cataract surgical complications	2.9%	0.0%	0.7%	0.4%
6. Non Trachomatous corneal opacity	1.4%	4.1%	7.0%	3.5%
8. Myopic retinopathy	8.7%	12.2%	4.9%	2.7%
9. Glaucoma	4.3%	0.0%	2.1%	1.3%
10. Diabetic retinopathy	2.9%	4.1%	2.1%	0.9%
11. ARMD	4.3%	18.4%	17.5%	13.3%
12. Other posterior segment disease	5.8%	10.2%	5.6%	4.9%
13. All other globe/CNS abnormalities^a^	2.9%	0.0%	2.1%	0.9%
Total	100.0%	100.0%	100.0%	100.00%
A. Treatable (1,2,3)	66.7%	51.0%	58.0%	72.1%
B. Preventable (PHC/PEC services) (5,6,7,8)	10.1%	16.3%	11.9%	6.2%
C. Preventable (Ophthalmic services) (4,9,10)	10.1%	4.1%	4.9%	2.7%
D. Avoidable (A + B + C)	87.0%	71.4%	74.8%	81.0%
E. Posterior segment causes (8,9,10,11,12)	26.1%	44.9%	32.2%	23.0%

^a^All other globe/CNS abnormalities: Microphthalmos, anophthalmos, enucleated eye, amblyopia

Our data showed that cataract surgical coverage in Altay and Tacheng among blind people was 64.7% and around 50% among people with SVI and MVI (Table 4).Statistical analysis showed no significant difference in these rates between Han Chinese and either Khazak or Uygur people or between male and female Khazaks or Uygurs. There was also no statistically significant difference found in terms of cataract surgical coverage rates based on distance from home to the two prefecture level hospitals in study areas where ocular services were delivered (Data not been presented).

**Table 4:** Cataract surgical coverage by visual acuity, gender and ethnicity (95% confidence interval)

	Sex(%)		Ethnicity(%)				
Visual Acuity	Male	Female	Han	Khazak	Uygur	Other	Total
VA < 3/60	61.3(42.2–78.2)	66.7(52.5–78.9)	71.8(55.1–85.0)	56.4(39.6–72.2)	100	60.0(14.7–94.7)	64.7(53.6–74.8)
VA < 6/60	50.0(34.2–65.8)	61.0(47.4–73.5)	57.1(42.2–71.2)	52.3(36.7–67.5)	100	60.0(14.7–94.7)	56.4(46.2–66.3)
VA < 6/18	40.7(27.6–55.0)	48.2(37.1–59.4)	46.9(34.3–59.8)	43.3(30.6–56.8)	75(19.4–99.4)	33.3(7.49–70.1)	45.3(36.7–54.0)

Table 5 shows the reported barriers to cataract surgery in people with moderate VI or worse. The most common barrier was lack of awareness that treatment was available (49.1%), Khazaks experienced significantly greater local barriers to seeking treatment (e.g. transportation, convenience) (*p* < 0.01). More women also reported that they could not access treatment [8] compared to men.

**Table 5:** Barriers to cataract surgery – bilateral PVA < 6/18 due to cataract, n (%), (each person can select one or two barriers)

	Male	Female	Han	Khazak	Uygur	Other	Total
	n(%)	n(%)	n(%)	n(%)	n(%)	n(%)	n(%)
Total	68	95	78	66	2	17	163
Need not felt	18 (26.5)	25 (26.3)	22 (28.2)	16 (24.2)	2 (100.0)	3 (17.7)	43 (26.4)
Fear	16 (23.5)	17 (17.9)	13 (16.7)	17 (25.8)	0 (0.0)	3 (17.7)	33 (20.3)
Cost	4 (5.9)	2 (2.1)	2 (2.6)	3 (4.6)	0 (0.0)	1 (5.9)	6 (3.7)
Treatment denied by provider	3 (4.4)	12 (12.6)	8 (10.3)	6 (9.1)	0 (0.0)	1 (5.9)	15 (9.2)
Unaware treatment is possible	36 (52.9)	44 (46.3)	42 (53.9)	26 (39.4)	1 (50.0)	11 (64.7)	80 (49.1)
Cannot access treatment	6 (8.8)	25 (26.2)*	13 (16.7)	17 (25.8)	0 (0.0)	1 (5.9)	31 (19.0)
Local reasons	7 (10.3)	9 (9.5)	2 (2.6)	13 (19.7)*	0 (0.0)	1 (5.9)	16 (9.8)

*P < 0.01

With available correction over half (54.5%) of all operated eyes had good outcomes, with VA equal or better than 6/12 and 20% of operated eyes had poor outcomes (VA worse than 6/60).

Table 6 showed that Han Chinese had greater proportion of people wearing glasses compared to the minority groups of people.

**Table 6:** Proportion of subjects wearing glasses, by ethnic group comparing Han with other groups, n (%)

	Han (*n* = 1712)		Khazak (*n* = 1747)		Uygur (*n* = 105)		Others (*n* = 413)		Total (*n* = 3977)	
Wearing glasses for any reason	642	37.5%	198	(11.3%)*	15	14.3%*	100	24.2%*	955	24.0%
Wearing distance glasses	59	3.5%	26	(1.5%)*	6	5.7%	5	1.2%**	96	2.4%
Wearing presbyopic glasses	592	34.6%	177	(10.1%)*	10	9.5%*	96	23.2%*	875	22.0%

* *p* < 0.001 ** *p* < 0.05

## Discussion

This survey has demonstrated the magnitude and causes of blindness, visual impairment in minority ethnic groups (Khazak, Uygur, Other) and Han Chinese in Altay and Tacheng, Xinjiang. We have found a low level prevalence of blindness (1.7%, 95% confidence interval: 1.4–2.2%), in this population comparing to prevalence rates identified through studies of other regions in China [4], with no evidence of inequalities between minority ethnic groups. People of Khazak origins reported greater difficulties in accessing cataract surgery due to local barriers such as lack of transport, and women also reported greater difficulties in accessing treatment.

The low prevalence of blindness found in this study was consistent with many other studies in China [4, 10–12]. This finding was consistent with that of neighboring countries such as Mongolia [13], and other central Asian Countries [14], although lower than South East Asia Countries [14–17].

Altay and Tacheng are located in relatively affluent part of Xinjiang and had better public health recognition, whereas in the south of Xinjiang, the situation was not so favorable. The local health authorities recommended future eye studies being conducted in South Xinjiang to better understand the need for eye health information and education campaigns and eye health services in Xinjiang.

We found a higher prevalence of blindness and visual impairment among Khazak people, though this was no statistically significant compared to Han Chinese. This result differs from the results of studies conducted in Yunnan [18] or Tibet [19], where the authors reported minority groups (Bai in Yunnan, and Tibetan in Tibet) had higher prevalence of vision problems than among the local Han Chinese, both studies examined the minority ethnic groups only and made inferences based on comparisons with published literature. In this study, however, we have examined all ethnic groups with the same teams and same methods and therefore our comparisons are likely more valid. We found that lower levels of EVI in Khazak people compared to the Han Chinese. The major cause of EVI was uncorrected refractive error. We also found that greater proportions of Han Chinese wared glasses compared to minority groups, especially Khazak people (Table 6), indicating lower prevalence of refractive error (RE) among minority people in Xinjiang. This could be due to lifestyle differences, where predominantly nomadic Khazak people spend more time outdoors than do the Han counterparts [20]. Further study on genetic analysis may also help to have better explanation on the differences.

This indicates a need to ensure adequate RE services are available to people living in Xinjiang, which would also benefit people who have had cataract surgery.

The most common cause of blindness was cataract, which is similar to other blindness surveys in China and other low and middle income countries [4, 15–17]. Similarly, the most common cause of VI was uncorrected refractive error. Myopic retinopathy was found the most common irreversible cause of blindness and VI in this study, which contrasts to data from other surveys, where glaucoma and corneal opacities are more common. Myopia is common in Chinese children and prevention strategies under investigation in China may have an impact on future cohorts [20]. However, this is the first time that myopic retinopathy has been documented as one of the most common cause of blindness in an older Chinese population although the Shipai Eye Study in Taiwan found that was the second cause of visual impairment [21].

Cataract surgical coverage (CSC) rates were similar across gender, but was lower amongst Khazak cataract blind (56.4%, 95%CI: 36.9–72.2%) compared to Han Chinese (71.8%, 95%CI: 55.1–85.0%) though the differences were again not statistically significant. The low numbers of people from Uyghur and other ethnic minority groups who were blind makes comparisons of CSC difficult. The most common barrier to cataract surgery was lack of awareness that treatment is possible, this is similar to findings from Jiangxi Province [12], Inner Mongolia [22], China.

Reported barriers to cataract surgery were similar for most causes between men and women, with the exception of access, where higher proportions of women reported difficulties. Higher proportions of Khazak were more likely to report local barriers such as transport.

There were limitations in this study. A higher proportion of Khazak people were not available for eye examination during the scheduled visit to their homes. This was because most Khazak people in the study were nomadic herders and could not be reached where at their registered address as they stayed with their livestock in pastureland during summer months when the study was conducted. This may have led to an over estimation of prevalence of blindness and an under estimation of prevalence of EVI. However, the overall participation rates were high for all ethnic groups (all > 95%) which limits the potential bias this could introduce.

There was also a low proportion of people of Uyghur origin, which is representative of the local population, but may not be generalization to the other parts of Xinjiang. Therefore, further studies in other parts of Xinjiang with larger Uyghur populations would be of benefit. The higher levels of barriers to access amongst Khazak suggest inequities in access though no evidence of inequalities in eye health outcomes, but statistically insignificant differences in prevalence of blindness. The small numbers limits the power of statistical tests, however, we have shown clear differences in perceptions of access, which will inform further planning of services.

There is also a need for increased eye health education, as indicated by the large number of participants who indicated they were unaware that cataract treatment is possible. Education on eye health and cataracts could also address other barriers such as feared and need not perceived. More women compared to men reported they could not access treatment and higher proportions of Khazak reported local reasons such as transport, although CSC was higher in women overall because they care more about their health situation according to the local health officials who reported that in many other health program, women had higher attendance rate than men. This suggests access to the patients in remote areas should be considered. These findings were consistent with the findings from other studies for cataract surgical outcome in China [23, 24] as some rural communities live in remote areas which are difficult to access.

## Conclusion

The study documented low blindness prevalence (1.7%, 95%CI 1.4–2.2%) among people at 50 years old and above comparing to prevalence rate estimated in other areas in China, but it still indicates blindness and un-operated cataract as the significant public health issue, with no significant differences in prevalence among minority groups of people in Xinjiang. Accessible eye health information and eye care will reduce inequities in barriers in access to cataract surgery between ethnic minority groups in Xinjiang.

## Abbreviations

BCVA: Best Corrected Visual Acuity
BL: Blindness
CI: Confidence Interval
CSC: Cataract Surgical Coverage
CSR: Cataract Surgical Rate
EVI: Early Visual Impairment
IAPB: International Association of Prevention of Blindness
MVI: Moderate Visual Impairment
PVA: Presenting Visual Acuity
RAAB: Rapid Assessment of Avoidable Blindness
RE: refractive Error
SVI: Severe Visual Impairment
URE: Uncorrected Refractive Error
VA: Visual Acuity
VI: Visual Impairment
WHO: World Health Organization
